# Supplementary material for: Reactive/Less-cooperative individuals advance population’s synchronization: Modeling of Dictyostelium discoideum concerted signaling during aggregation phase
Source: PLoS One. 2021 Nov 18;16(11):e0259742. doi: 10.1371/journal.pone.0259742 (PMC8601469; doi:10.1371/journal.pone.0259742)
Supplement: S1 Appendix — (PDF) [file pone.0259742.s001.pdf]

S1 Appendix. Numerical simulation and data analysis As mentioned above, we perform numerical experiments using the slightly modified Barkley model [1]. We simulate the system of Eq.1 using the finite difference method:

$$\frac{\partial u_{ij}}{\partial t} = \nabla^2 u_{ij} + u_{ij}(1 - u_{ij})(u_{ij} - \frac{v_{ij} + b}{a}), \tag{3}$$

$$\frac{\partial v_{ij}}{\partial t} = \epsilon(u_{ij} - v_{ij}) + \eta_{ij}(t). \tag{4}$$

As illustrated in Fig.1-A, the boundary layer with size δ defines the threshold value for u_{ij}.We assume that $u = 0$ within the interval $[0.\delta)$,. i. e. in every cell whenever $u_{ij} < \delta$, its value in the next update will be considered equals to zero. The values of the variables are updated using a discrete-time derivative Euler method. Here, we compute ∇^2 as the discrete Laplace operator of a $2D$ variable on the grid, using a five-point stencil finite difference method as $\nabla^2 u_{ij} = \frac{1}{h^2}(u_{i+1j} + u_{i-1j} + u_{ij-1} - 4u_{ij})$ where h is the grid spacing. We perform our simulations on a 400×400 lattice with Neumann boundary condition state that the spatial derivatives with respect to the normal vectors are null on the boundaries of the domain. We implement these boundary conditions by duplicating values in matrices u and v on the edges at each time step. Throughout this study, the physical values are set as $a = 0.3$, $b = 0.01 \, and \, \epsilon = 0.005$. Besides, numerical values are adjusted as $h = 0.25$, $\Delta t = 0.05 \, and \, \delta = 0.0125$. The parameters are regulated to yield the network in a sub-excitable state, i.e., the formation of sustainable spatial patterns is impossible without the existence of η term. Parallel with the integration of the deterministic part, we implement the Euler–Maruyama technique with a time step Δt to integrate the $\eta(t)$ stochastic dynamics,

$$\eta_{ij}(t + \Delta t) = \eta_{ij}(t) - \lambda \eta_{ij}(t)\, \Delta t + \lambda \sqrt{2\,D} \Delta t \, \Delta W_{ij}(t) \tag{5}$$

where the $\Delta W(t)$ are independent Gaussian random numbers with unit variance (known as increments of the Wiener process [2]). Stochastic values are set as $D = 1$ and $\lambda = 1/300$. Considering them as the transient period, signals occurring in the first 900 time steps are excluded from the calculations. Besides, the edges of the lattice is removed in the analysis to avoid artifacts from the event recognition at the borders. In addition, there are 100 randomly distributed pixels that fire with a random amplitude between 0.01 and 0.02 during the entire simulations.

References

1. Barkley D. A model for fast computer simulation of waves in excitable media. Physica D. 1991;49:61–70.

2. Laing C, Lord CJ. Stochastic Methods in Neuroscience, Oxford University Press, New York; 2010.
